# The Situation

**Published:** 1896-09

**Authors:** 


### EDITORIAL DEPARTMENT.

F. E. DANIEL, M. D., Editor.
S. E. HUDSON, M. D., Managing Editor.
A. J. SMITH. M. D., Galveston, Associate Editor.

EDITORIAL STAFF:

PROF. J. E. THOMPSON, M. D., Texas Medical College, Galveston; Surgery.
PROF. WM. KEILLER, M. D., Texas Medical College, Galveston; Obstetrics and Gynecology.
PROF. DAVID CERNA, M. D., Texas Medical College, Galveston; Therapeutics.
PROF. A. J. SMITH, M. D., Texas Medical College, Galveston; Medicine.
DR. R. H. L. BIBB, City of Mexico; Foreign Correspondent.

Published Monthly at Austin, Texas, by Drs. Daniel and Hudson. Subscription price $2.00 a year in advance.

Eastern Agents: The Monihan-Fairchild Special Agency, 24 Park Place, New York City.

Official organ of the West Texas Medical Association, the Houston District Medical Association, the Austin District Medical Society, the Galveston County Medical Society, and several others.

### EDITORIAL.

THE SITUATION:—It appears from the letter of Prof. Donald Maclean, published elsewhere in this issue, that the same embarrassing situation with reference to medical legislation exists in Michigan as in Texas; and that almost identical steps are being taken to solve the difficulty. It seems that Michigan, like Texas, is infested with irregulars; and that the regular profession have, for years, vainly attempted to secure proper legislation for the "regulation of the practice," and that they have, in this extremity, committed, or are trying to commit, the same absurdity that is proposed in Texas:—i. e., to *unite with existing irregulars to legislate against future irregularity;* the exception being, and it is a notable one, that Dr. Maclean who, for years, has been at the head of the legislative committee of the regular profession, repudiates the action now proposed, while in Texas those who have been most conspicuously connected with our past efforts (and failures), are the very ones to offer this compromise and to champion it.

Dr. Maclean's letter is good reading. His arguments cannot be answered. They apply as well to Texas as to Michigan. I commend his letter to the thoughtful consideration of our readers, and trust they will do all in their power to save the profession of Texas and the Texas State Medical Association from the consequences of this humiliating concession to homeopathy.*

TO THE WRITER there is something so supremely absurd and ridiculous in the proposition to invoke the aid and assistance of acknowledged irregulars in an effort *to legislate against irregularity* that he cannot bring himself to contemplate it with equanimity, or to even treat the subject with becoming dignity.

We have said, however, all we intend to say upon this subject. It is no funeral of ours—and we are not the guardian of the profession's interest, honor or dignity. We have exercised the right and privilege of a journalist to express our opinion on the subject, and advise against the proposed action. If the State Medical Association sees proper to humble itself in this way for the accomplishment of its desire,—to protect the people from quackery by and with the aid of a part of the quacks; or, if it sees proper to throw ethics to the wind; to go back on its record; to falsify its enunciations made at Galveston in 1877 and elsewhere up to date; to abandon all principle to a mistaken notion of expediency; and thus to falsify one of the declarations of its objects in organizing—"to encourage a high standard of professional qualifications and *ethics*,"—why, let them. We will simply look on with regret, and "deplore the degeneracy of the age."

We would respectfully suggest, however, that before this affiliation and joint effort with irregulars is tried, it might be well to consider whether it might not be better and easier of accomplishment to get the existing law amended so as to answer the purpose. The legislature has shown itself very reluctant to legislate on the subject at all, and it appears reasonable to suppose that it would be easier to get an amendment through, than an entirely new bill.

It will be remembered that in 1876, or 1879, when the bill which is now the law in force, was going through the legislature, it provided, in effect, that all persons hereafter offering to practice should be examined by the district boards provided for in the bill. When it was on its last reading in the senate, an old doctor, MacCollum, who was a senator, got in an amend-

*The senior editor writes in the first person, because his associate, the junior editor and business manager, is not in accord with the policy advocated by him.—F. E. D.

ment about in these words, "or have a diploma from some regularly chartered medical college." The amendment prevailed, and has had the effect of destroying the virtue of the bill. We respectfully suggest that it is worth trying to see if an amendment cannot be gotten through, striking out Dr. McCollum's emasculating clause. This would leave the bill in a very satisfactory condition; and we trust those who have charge of the subject of legislation will consider it.

